# Diacerein retards cell growth of chondrosarcoma cells at the G2/M cell cycle checkpoint via cyclin B1/CDK1 and CDK2 downregulation

**DOI:** 10.1186/s12885-015-1915-4

**Published:** 2015-11-10

**Authors:** Birgit Lohberger, Andreas Leithner, Nicole Stuendl, Heike Kaltenegger, Werner Kullich, Bibiane Steinecker-Frohnwieser

**Affiliations:** Department of Orthopedic Surgery, Medical University Graz, Auenbruggerplatz 5, A-8036 Graz, Austria; Ludwig Boltzmann Institute for Rehabilitation of Internal Diseases, Ludwig Boltzmann Cluster for Rheumatology, Balneology and Rehabilitation, Saalfelden, Austria

**Keywords:** Chondrosarcoma, Diacerein, Cell cycle arrest, Cyclin B1, CDK

## Abstract

**Background:**

Chondrosarcoma is characterized for its lack of response to conventional cytotoxic chemotherapy, propensity for developing lung metastases, and low rates of survival. Research within the field of development and expansion of new treatment options for unresectable or metastatic diseases is of particular priority. Diacerein, a symptomatic slow acting drug in osteoarthritis (SYSADOA), implicates a therapeutic benefit for the treatment of chondrosarcoma by an antitumor activity.

**Methods:**

After treatment with diacerein the growth behaviour of the cells was analyzed with the xCELLigence system and MTS assay. Cell cycle was examined using flow cytometric analysis, RT-PCR, and western blot analysis of specific checkpoint regulators. The status for phosophorylation of mitogen-activated protein kinases (MAPKs) was analyzed with a proteome profiler assay. In addition, the possible impact of diacerein on apoptosis was investigated using cleaved caspase 3 and Annexin V/PI flow cytometric analysis.

**Results:**

Diacerein decreased the cell viability and the cell proliferation in two different chondrosarcoma cell lines in a dose dependent manner. Flow cytometric analysis showed a classical G2/M arrest. mRNA and protein analysis revealed that diacerein induced a down-regulation of the cyclin B1-CDK1 complex and a reduction in CDK2 expression. Furthermore, diacerein treatment increased the phosphorylation of p38α and p38β MAPKs, and Akt1, Akt2, and Akt 3 in SW-1353, whereas in Cal-78 the opposite effect has been demonstrated. These observations accordingly to our cell cycle flow cytometric analysis and protein expression data may explain the G2/M phase arrest. In addition, no apoptotic induction after diacerein treatment, neither in the Cal-78 nor in the SW-1353 cell line was observed.

**Conclusions:**

Our results demonstrate for the first time that the SYSADOA diacerein decreased the viability of human chondrosarcoma cells and induces G2/M cell cycle arrest by CDK1/cyclin B1 down-regulation.

## Background

Diacerein represents a symptomatic slow acting drug in osteoarthritis (SYSADOA) from the anthraquinone chemical class and as the general term implicates, efficacy against the symptoms of osteoarthritis (OA) has been demonstrated [1]. As an anti-rheumatic drug, the potential of diacerein lies in the *in vitro* inhibition of the synthesis of interleukin-1 and its activity within the synthesis of proteoglycans, glycosaminoclycans, and hyaluronuic acid, principle components of cartilage extracellular matrix [2]. By using an experimental canine model of OA, an effective reduction in chondrocyte DNA fragmentation and cell death, due to a diacerein induced reduction of caspase-3 activity has been observed [3]. Within the early lesions of experimental OA the activation of the caspase cascade has been connected to chondrocyte death, whereas caspase as well as MEK1/2 and p38MAPK inhibitors reveal a marked deterioration of the programmed cell death and attenuate the severity of cartilage lesions [4, 5]. Studying the cell proliferation and cell viability characteristics of C28/I2 chondrocytes, strikingly increased concentrations of diacerein significantly decreases cell growth and viability [6]. These observed growth-inhibiting qualities of diacerein, when applied at higher concentrations, might implicate a therapeutic benefit for the treatment of chondrosarcoma [7]. While diacerein has proved to be effective in the treatment of OA, Qin et al described that a diacerein α-aminophosphonate conjugate has anti-proliferative activities on tumor cells [8].

Chondrosarcomas constitute a heterogeneous group of neoplasms, tumor cells with the common characteristics in terms of the production of components of the extracellular matrix within the cartilage [9]. With an incidence of 1:50,000, chondrosarcoma typically occurs in adults in their 3^rd^ to 6^th^ decade of life and represent the second most common primary malignant bone tumor in large epidemiologic series [10]. Wide surgical excision remains the best available treatment for intermediate- to high-grade tumors since they are relatively chemo- and radiotherapy resistant because of their extracellular matrix, low percentage of dividing cells, and poor vascularity, [11–14]. However, for high-grade chondrosarcoma, the prognosis is poor even after adequate surgery [15]. From the clinical point of view it is a huge challenge within the field of cancer treatment, to prevent recurrence and to find better treatment options for unresectable or metastatic diseases, such as chondrosarcoma.

The aim of this study was to show if diacerein is able to generate a reduction in cell growth and if this decline is generated by cell cycle arrest or apoptosis. Therefore, the effect of diacerein on cell proliferation, cell cycle distribution, and apoptosis of two human chondrosarcoma cell lines was investigated.

## Methods

### Cell culture

Human chondrosarcoma cell lines SW-1353 (CLS, Eppelheim, Germany) and Cal-78 (DSMZ, Braunschweig, Germany) were cultured in Dulbecco’s-modified Eagle’s medium (DMEM-F12; GIBCO®, Invitrogen, Darmstadt, Germany), containing 5 % fetal bovine serum (FBS), 1 % L-glutamine, 100 units/ml Penicillin, 100 μg/ml Streptomycin, and 0.25 μg Amphotericin B (all GIBCO®, Invitrogen). Both cell lines were verified by short tandem repeat analysis using PowerPlex 16 System Kit (Promega, Mannheim, Germany). Cells were kept at 37 °C in a humidified atmosphere of 5 % CO_2_ and were passaged by trypsinization after reaching 80–90 % confluence.

### Sample preparation

Pure Diacerein (TRB Chemedica International, Geneva, Switzerland) was dissolved in DMSO and diluted with culture medium. The final DMSO concentration was max. 0.5 %, which did not affect the behavior of the cells as observed by benchmark experiments.

### Cell proliferation

*MTS assay* (Brand, Voerde-Friedrichsfeld, Germany) was used to measure the metabolic activity of cells: 5 × 10^3^ cells per well were seeded into 96 well plates and treated with 0–500 μM diacerein. The cells were treated for 24 h and 48 h, thereafter a CellTiter 96 AQueous Assay (Promega, Mannheim, Germany) was performed following the manufacturers’ instructions; untreated cells were used for control (ctrl).

The *xCELLigence DP* device from Roche Diagnostics (Mannheim, Germany) was used to monitor cell proliferation in realtime after cells were seeded on electronic microtiter plates (E-Plate; Roche Diagnostic) [16]. Cells were treated with 0, 30, 100, and 300 μM diacerein and the proliferation rate was measured for 24 h. Cell index (CI) measurements were performed in triplicates with a signal detection set for every 20 min. The cell index (CI) is a measure for the cell density of cells and was normalized to the time point when diacerein was added. Subsequent to the continuous xCELLigence cell monitoring, the slope (1/h) representing the rate of change of the cell index was calculated from time 7–24 h. Acquisition and analysis was performed with the RTCA software (Version 1.2, Roche Diagnostics).

### Flow cytometry for cell cycle analysis

SW1353 and Cal-78 cells were treated with 30, 100, and 300 μM diacerein. After a treatment time of 24 and 48 h, respectively, the cells were harvested by trypsinization and fixed with 70 % ice-cold ethanol for 10 min at 4 °C. Next to washing, the cell pellet was re-suspended in PI-staining buffer (50 μl/ml PI, RNAse A, Beckman Coulter, Brea, CA) and was incubated for 15 min at 37 °C. Cell cycle distribution was analyzed by FACS Calibur (BD Biosciences, San Diego, CA) using ModFit software.

### Reverse transcription polymerase chain reaction (RT-PCR)

Total ribonucleic acid (RNA) was isolated from treated and untreated cells with the RNeasy Mini Kit and DNase-I treatment according to the manufacturer’s manual (Qiagen, Hilden, Germany). One microgram RNA was reverse transcribed using a RevertAid cDNA Synthesis Kit (Fermentas). Amplification was achieved with the Platinum SYBR Green Super Mix with ROX (Invitrogen) on AB7900HT (Applied Biosystems, Invitrogen). Each qPCR run consisted of a standard 3-step PCR temperature protocol (annealing temperature of 60 °C) followed by a melting curve protocol to confirm a single gene-specific peak and to detect primer dimerization. Relative quantification of expression levels were obtained by the ∆∆Ct method based on the geometric mean of the internal controls glyceraldehyde 3-phosphate dehydrogenase (GAPDH), β-actin (ACTB), and hypoxanthine phosphoribosyl-transferase (HPRT-1), respectively. The following primers were used for real time RT-PCR: QuantiTect primer assays (Qiagen) for cyclin B1 (ID QT00006615), CDK1 (ID QT00042672), and CDK2 (ID QT00005586). The expression level (C_T_) of the target gene was normalized to the reference genes (ΔC_t_), the ΔC_t_ of the test sample was normalized to the ΔC_t_ of the control (ΔΔC_t_). Finally, the expression ratio was calculated with the 2^-ΔΔCt^ method (*p < 0.05).

### Western blot analysis

For immunoblotting, whole cell protein extracts were prepared with lysis buffer (50 mM Tris–HCl pH 7.4, 150 mM NaCl, 50 mM NaF, 1 mM EDTA, 10 % NP-40, 1 % Triton-X, and protease inhibitors), subjected to SDS-PAGE (10 or 12 %) and blotted onto PVDF membrane (Roth, Karlsruhe, Germany). Primary antibodies against cyclin B1 (sc-245), CDK1 (sc-54), CDK2 (sc-6248), and p53 (sc-126) were purchased from Santa Cruz (Santa Cruz Biotechnology, Santa Cruz, CA), phospho-Histone H2A.X (#9718), Akt (#9272), phospho-Akt (Ser473) (#9271), p38 MAPK (#9212), and phospho-p38 MAPK (Thr180/Tyr182) (#4511) antibodies from Cell Signaling Technology (Cell Signaling Technology, Danvers, MA) and β-actin (A4700) from Sigma-Aldrich (Vienna, Austria). Blots were developed using horseradish peroxidase-conjugated secondary antibodies (Dako, Jena, Germany) at room temperature for 1 h and the SuperSignal® West Pico Chemoluminescent Substrate (Thermo Scientific, Rockford, IL), in accordance with the manufacturers’ protocol.

### MAPK array

For the Proteome Profiler Human Phospho-MAPK Array Kit (ARY002B; R&D Systems, Minneapolis; MN), cells were treated with 30 μM diacerein for 24 h and whole cell protein extracts were prepared with lysis buffer. Capture and control antibodies of the major families of mitogen-activated protein kinases (MAPKs), the extracellular signal-regulated kinases (ERK1/2), c-Jun N-terminal kinases (JNK1-3), and different p38 isoforms (α/β/δ/γ), have been spotted in duplicate on nitrocellulose membranes. To analyzing the phosphorylation status the array was performed following the manufacturers’ instructions; untreated cells were used for control.

### Caspase-3 apoptosis assay

After incubation with with 30, 100, and 300 μM diacerein for 48 h, cells were harvested by trypsinization, fixed with formaldehyde for 10 min at 37 °C (2 × 10^6^ cells/ml), permeabilized with methanol, and finally re-suspended in incubation-buffer (FBS:PBS 1:200). Caspase-3, a marker for cells that undergo apoptosis, is activated by the proteolytic processing of its inactive zymogen into the activated p17 and p12 fragments, respectively. The FITC-conjugated monoclonal cleaved caspase-3 (Asp175) antibody (#9661; Cell Signaling Technology) detects endogenous levels of the large fragment (17/19 kDa), released after the activation of caspase-3. The antibody does not recognize full length caspase-3 or other cleaved caspases. Cells were analyzed by flow cytometry (FACS Calibur, BD Biosciences) performed with FACSDiva software. Histograms were created using FCS3 express software (De Novo software, Los Angeles, CA). Untreated cells were used as negative control.

### Annexin V/PI apoptosis assay

The APC Annexin V Apoptosis Detection Kit (BioLegends, San Diego, CA) was performed following the manufacturers’ instructions. Apoptotic cells were identified by the incubation of 1 × 10^5^ cells in 100 μl Annexin V binding buffer containing 5 μl Annexin V-APC and 5 μl PI for 15 min at room temperature. Flow cytometry analysis was performed with FACS Calibur (BD Biosciences); 10,000 events were collected. Cells were identified in the side scatter and forward scatter with linear scale. Fluorescence signals were shown with logarithmic scale. Compensation was performed by single Annexin and PI measurements and analyzed by FCS3 express software (De Novo software). Untreated cells were used as negative control.

### Statistical analysis

All values are expressed as mean values ± SD. Student’s unpaired *t*-test was used to evaluate differences between treated groups and their respective controls. The significance of dose or time responses was assessed by repeated measures analysis. Graphic data were prepared and calculated with SigmaPlot® (Systat Software Inc., San Jose, CA).

## Results

### Diacerein reduced cell proliferation and viability of chondrosarcoma cells

**Fig. 1:**
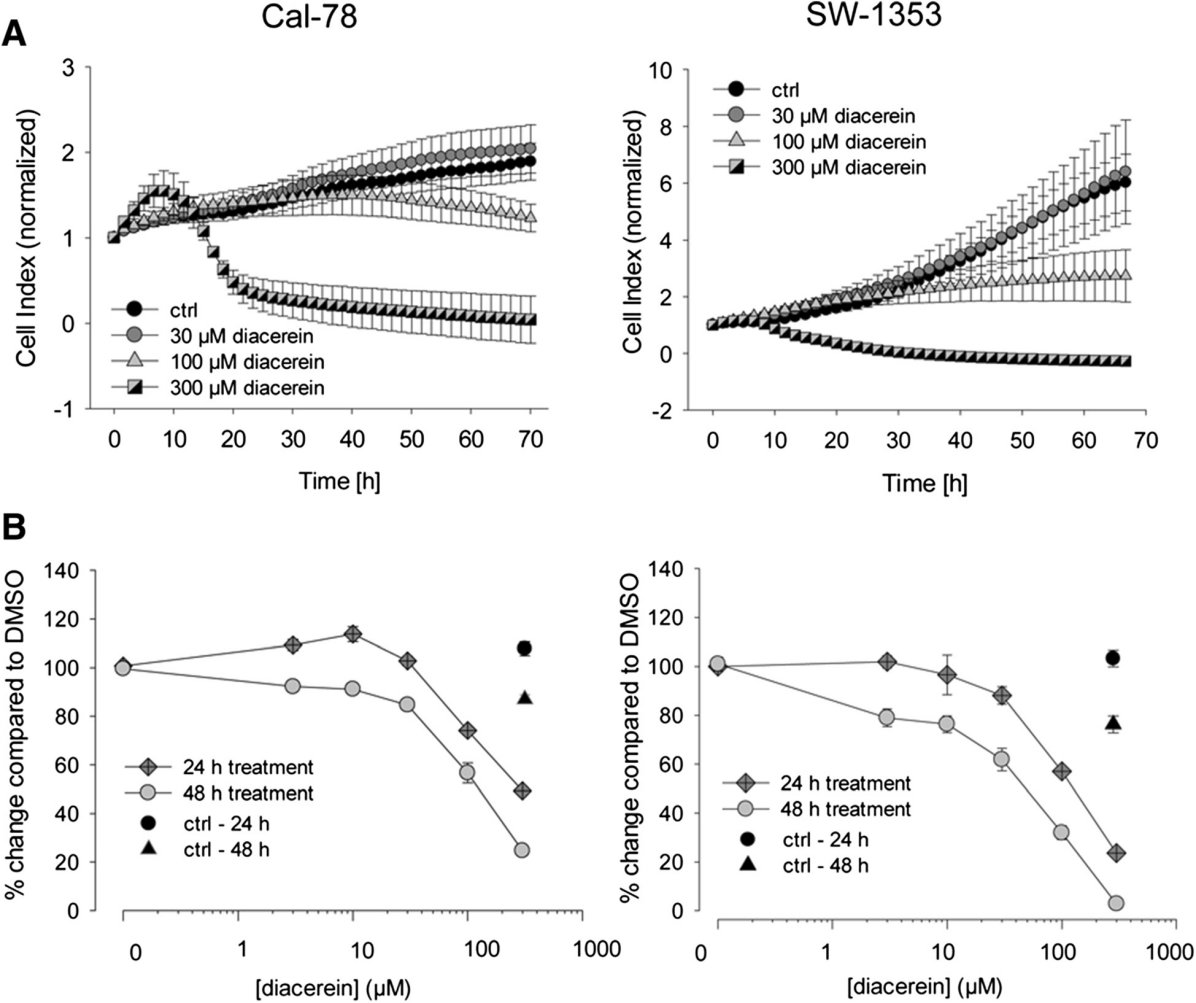
Influence of diacerein on cell viability and cell proliferation of chondrosarcoma cells. **a** Dynamic proliferation curves for Cal-78 and SW-1353 cells in the presence of 0 (*black circles*), 30 μM (*grey circles*), 100 μM (*light grey triangles*), and 300 μM (*bicolour squares*) diacerein. Data shown are representatives from three independent experiments (*n* = 3, measured in duplicates). **b** After incubation over 24 and 48 h diacerein inhibited cell growth in a concentration dependent manner. Untreated cells were measured as controls (*n* = 12, mean ± S.D.)

Chondrosarcoma cells were treated with 30, 100, and 300 μM diacerein for 48 h. During this period, cell growth curves were automatically recorded in real time by the xCELLigence System (Fig. 1a). Diacerein inhibited cell growth in a concentration dependent manner in both cell lines. While an effect on Cal-78 cells could only be observed at a concentration of 300 μM diacerein, causing a complete reduction of cell proliferation, SW-1353 cells demonstrated a higher sensitivity for diacerein illustrated by a considerably reduced cell index at a concentration of 30 and 100 μM. A block of cell growth or rather an induced cell death may contribute to this decrease in cell index. In Cal-78 cells slope values changed under the influence of diacerein from 0.022 ± 0.007 (ctrl) to 0.025 ± 0.006 (30 μM), -0.007 ± 0.016 (100 μM; *p* = 0.001), and −0.035 ± 0.007 (300 μM; p = 8.77E-11). In SW-1353 cells a change from 0.144 ± 0.026 (ctrl) to 0.166 ± 0.082 (30 μM), 0.028 ± 0.055 (100 μM; *p* = 0.012), and −0.028 ± 0.013 (300 μM; *p* = 1.01E-10) could be observed.

To investigate the influence on cell viability, chondrosarcoma cell lines were exposed to 0, 3, 10, 30, 100, and 300 μM of diacerein for 24 and 48 h. After the equivalent treatment, cells were measured by the MTS assay (*n* = 12). Figure 1b shows the time- and dose-dependent inhibition of cell viability.

### Diacerine caused a cell cycle G2/M arrest

**Fig. 2:**
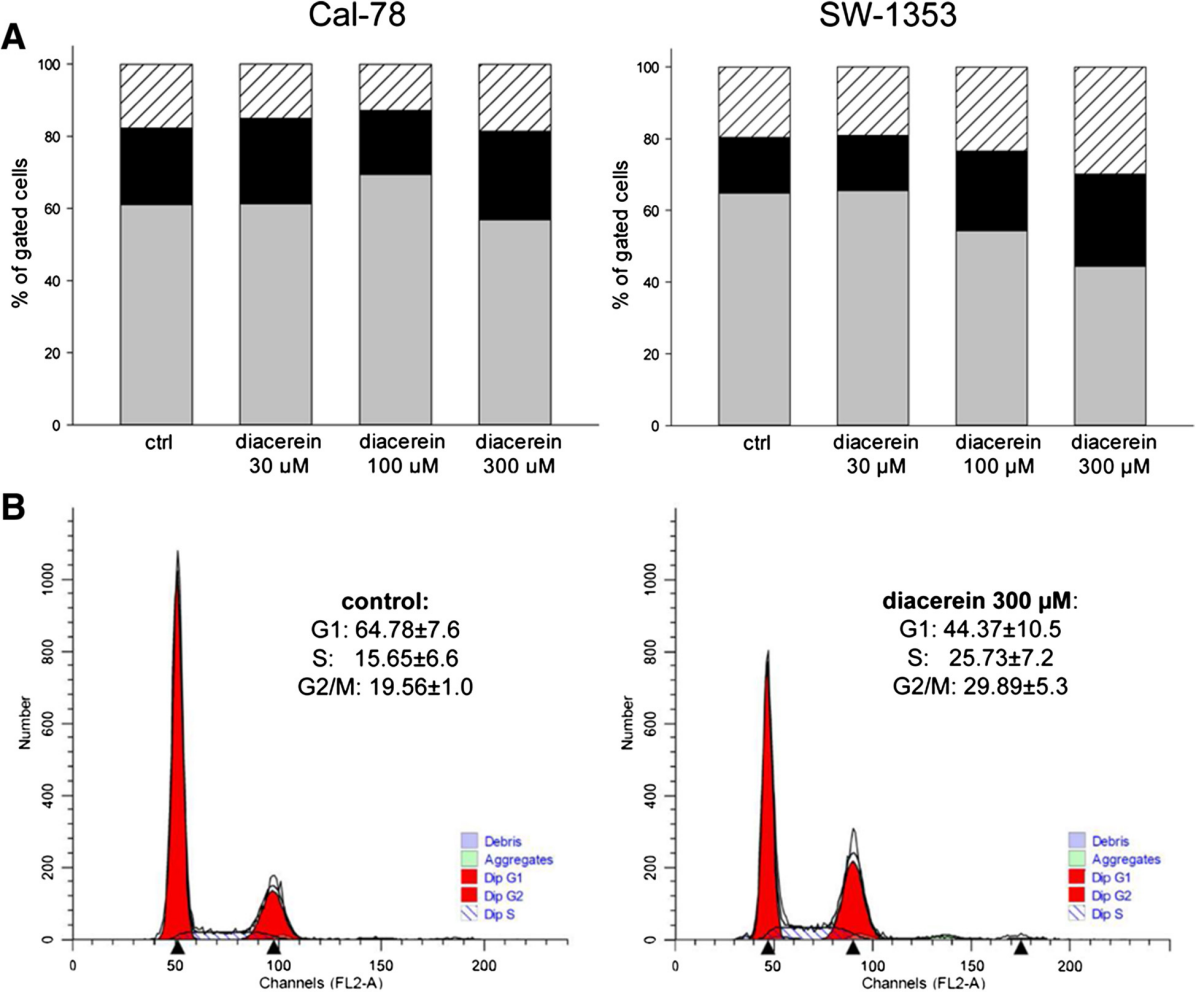
Graphical presentation of the cell cycle distribution. **a** Treatment of SW-1353 cells with 100 and 300 μM diacerein caused a significant decrease in the number of cells in G1 phase (*grey*) after 48 h which was accompanied by a pronounced increase of cells in S phase (*black*) and G2/M phase (*dashed lines*), while only small changes were observed in Cal-78 cells. Values are expressed as percentage of the cell population in the G1, S, and G2/M phase of cell cycle. **b** Representative measurements of untreated control and 300 μM diacerein treated SW-1353 cells

To investigate the effects of diacerein on cell cycle, chondrosarcoma cells were exposed to 30, 100, and 300 μM diacerein. Untreated cells were measured as controls. The cell cycle distribution for both cell lines (Cal-78 and SW-1353) is summarized in form of the stacked bar chart given in Fig. 2a. In SW-1353 cells, diacerein caused after 48 h exposure a pronounced decrease in the number of cells in the G1 (grey bars) phase, accompanied by a significant increase of the number of S (black bars) and G2/M phase (striated bars) cells, indicating a G2/M arrest. In the Cal-78 cell line only a moderate tendency towards G2/M phase could be observed. Representative measurements of untreated (control) and diacerein treated SW-1353 cells are depicted to highlight the differences (Fig. 2b). All values of three individual experiments (% of gated cells) are listed in Table 1.

**Table 1:** Cell cycle distribution of chondrosarcoma cell lines after 24 and 48 h exposure to different concentrations of diacerein^a^

Cell line	Treatment	h	G0/G1 (%)	S (%)	G2/M (%)
Cal-78	control	24 h	57.48 ± 8.19	23.79 ± 5.84	18.73 ± 2.51
	diacerein 30 μM	24 h	57.57 ± 8.30	25.23 ± 4.69	17.22 ± 3.68
	diacerein 100 μM	24 h	62.04 ± 5.54	22.01 ± 2.64	15.94 ± 2.85
	diacerein 300 μM	24 h	54.15 ± 7.33	26.76 ± 8.21	19.08 ± 3.67
	control	48 h	61.08 ± 3.03	21.23 ± 2.31	17.68 ± 0.89
	diacerein 30 μM	48 h	61.31 ± 1.16	23.66 ± 2.55	15.03 ± 1.80
	diacerein 100 μM	48 h	69.42 ± 2.80*	17.79 ± 2.38	12.78 ± 3.92
	diacerein 300 μM	48 h	56.95 ± 12.37	24.51 ± 9.02	18.53 ± 4.09
SW-1353	control	24 h	51.14 ± 7.05	22.73 ± 6.01	26.12 ± 1.35
	diacerein 30 μM	24 h	49.05 ± 5.46	27.15 ± 6.08	23.79 ± 1.23
	diacerein 100 μM	24 h	55.95 ± 4.11	19.53 ± 6.81	24.52 ± 6.83
	diacerein 300 μM	24 h	45.32 ± 12.41	25.35 ± 8.41	29.33 ± 7.06
	control	48 h	64.77 ± 7.67	15.65 ± 6.65	19.56 ± 1.03
	diacerein 30 μM	48 h	65.54 ± 5.75	15.37 ± 5.80	19.08 ± 0.31
	diacerein 100 μM	48 h	54.36 ± 5.92	22.16 ± 10.54	23.47 ± 4.86
	diacerein 300 μM	48 h	44.38 ± 10.52*	25.73 ± 7.23	29.89 ± 5.33*

^a^Asterisks represent significant differences between control and treated cells (*p* < 0.0.5; *n* = 3; mean ± S.D.)

### Diacerein decreased cyclin B1, CDK1, and CDK2 levels

**Fig. 3:**
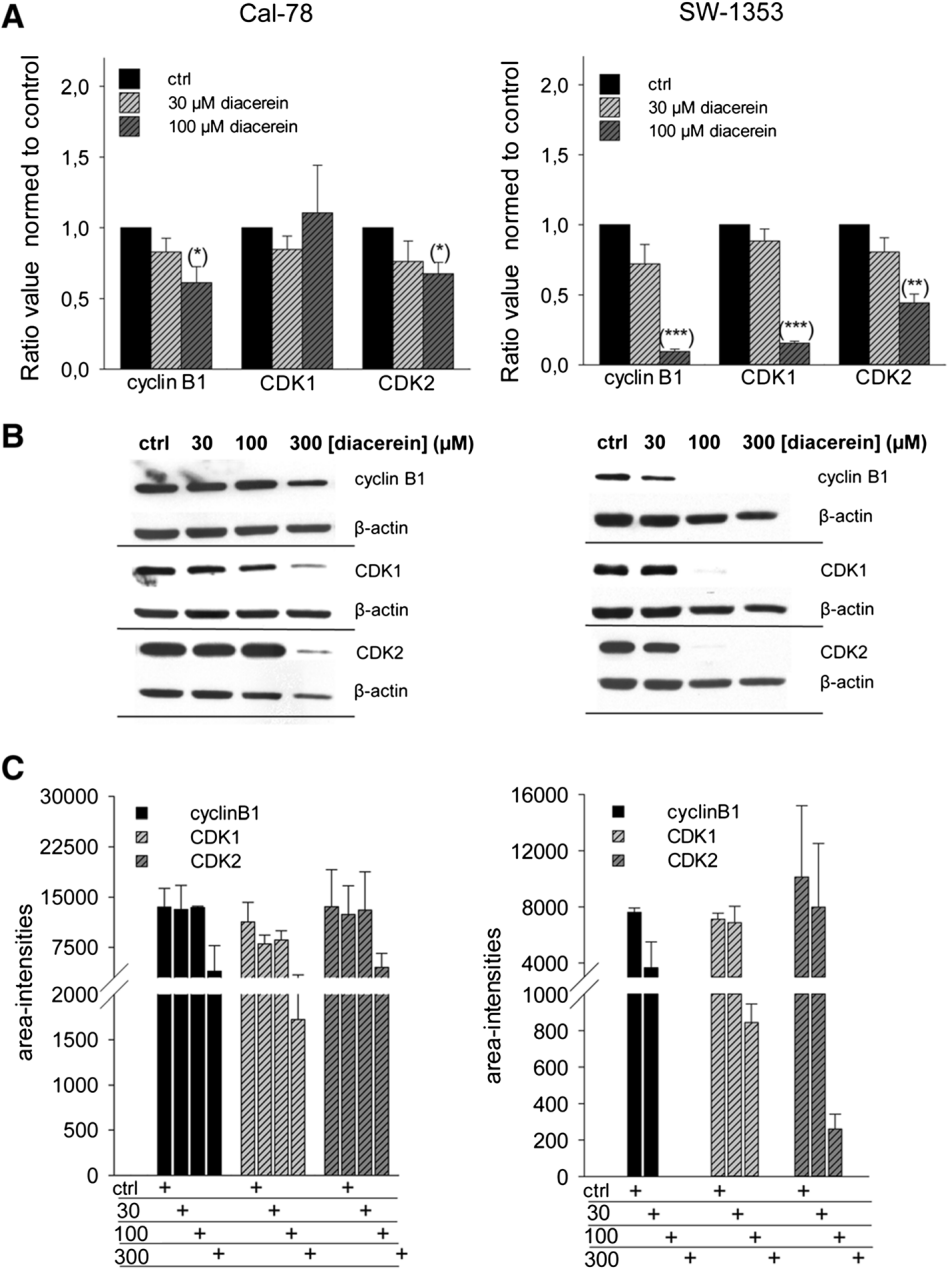
Regulation of the cell cycle checkpoints. **a** Relative mRNA expression of the cell cycle regulators cyclin B1, CDK1, and CDK2 after 48 h incubation with 30 and 100 μM diacerein. Asterisks represent significant differences between control and treated cells (* *p* < 0.05; ** *p* < 0.01; *** *p* < 0.001). **b** Total protein analysis after 48 h of treatment revealed a significant down-regulation of the G2/M arrest regulator proteins cyclin B1, CDK1, and CDK2 in 100 and 300 μM diacerein treated chondrosarcoma cells. β-actin was used as loading control. **c** Quantitative evaluation of the western blot analysis of cell cycle regulator proteins

Relative mRNA expression levels of cyclin B1, CDK1, and CDK2 were analyzed by real time RT-PCR after treatment of 30 and 100 μM diacerein for 48 h (Fig. 3a). Untreated control cells served as reference value (ratio = 1). In the case of Cal-78 cells treatment with diacerein (100 μM) induced a small, but not significant change, for the expression of CDK1, whereas the cyclin B1 and CDK2 levels were significantly down-regulated (cyclin B1: 0.612 ± 0.22 (*p* = 0.039); CDK2: 0.673 ± 0.16 (*p* = 0.027)) within the observation period. In SW-1353 cells a highly significant down-regulation by 100 μM diacerein in the expression of cyclin B1 to 0.095 ± 0.03 (*p* = 1.28E-05), CDK1 levels to 0.154 ± 0.02 (*p* = 9.82E-06), and the CDK2 levels to 0.443 ± 0.12 (*p* = 0.002) could be demonstrated.

In order to substantiate our observations, western blot analysis for the specific regulatory proteins responsible for the G2/M transition under the exposure of 30, 100, and 300 μM diacerein for 48 h were performed (Fig. 3b). Corresponding to the real time RT-PCR data, the expression of cyclin B1 and its corresponding cyclin-dependent kinases CDK1 and CDK2 were clearly declined in diacerein treated SW-1353 cells, whereas diacerein affected the expression of these G2/M checkpoint regulator proteins in Cal-78 cells only to a small extent. Figure 3c represents the quantitative evaluation of the western blot analysis of three independent experiments. All values were normalized to their corresponding β-actin.

### Phosphorylation of MAPKs under diacerein treatment

**Fig. 4:**
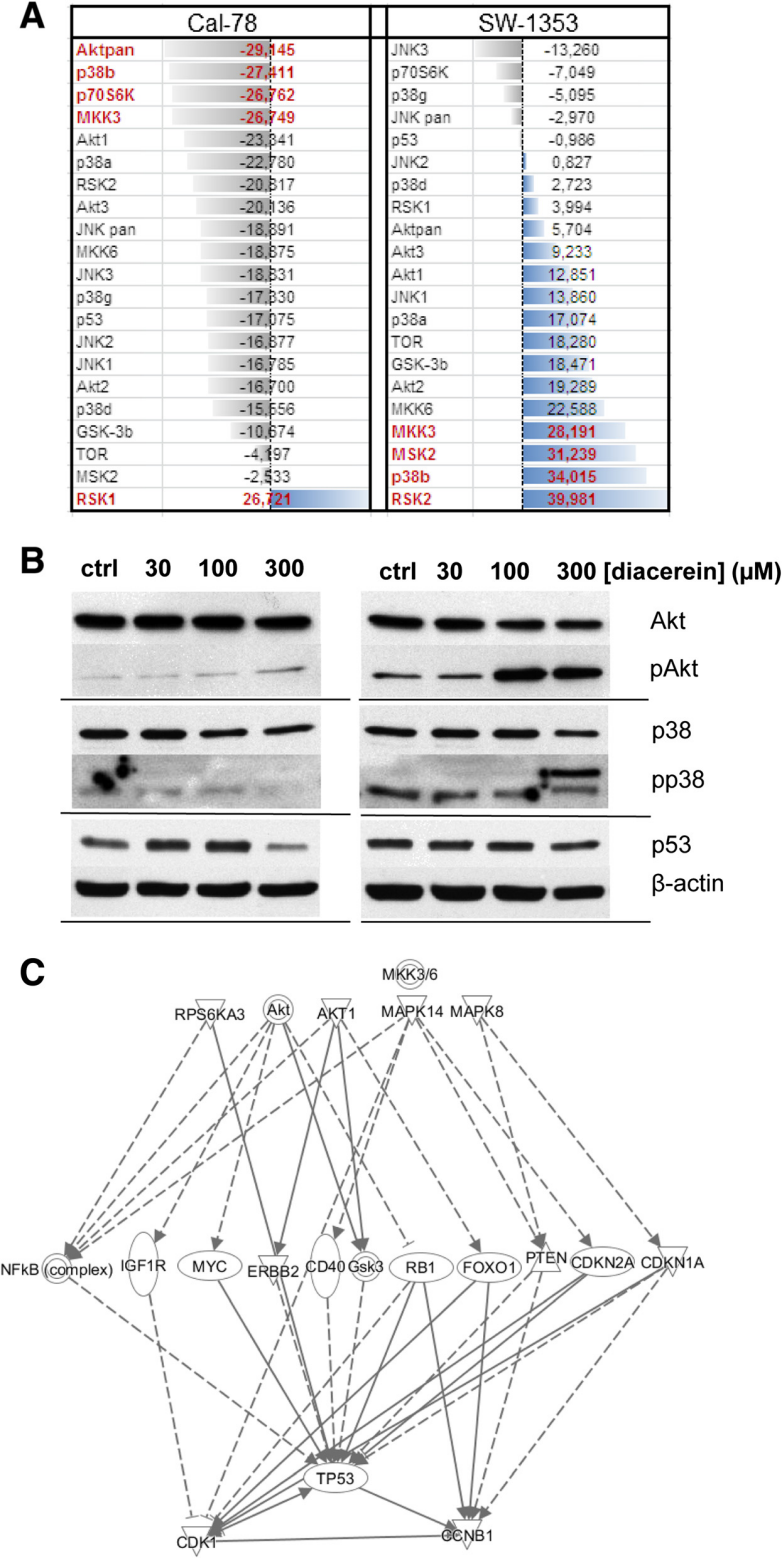
Phosphorylation of MAPKs under diacerein treatment. **a** MAPKs phosphorylation of Cal-78 and SW-1353 chondrosarcoma cell lines in percent change after 30 μM diacerein for 24 h. **b** Western blot analysis confirmed the results of the proteome profiler phospho-MAPK array and showed a significant increase of phospho-Akt and phospho-p38 after the treatment with different concentrations of diacerein in SW-1353 cells, whereas, no significant changes can be observed in the Cal-78 cells. The expression of p53 was diminished in Cal-78 cells. **c** Relationship of interesting genes using IPA (Ingenuity Pathway Analysis)

Treatment with 30 μM diacerein for 24 h increased the phosphorylation of p38α (17.07 % change) and p38β (34.01 %) MAPKs in SW-1353 cells, whereas in Cal-78 the observed phosphorylation changed to the contrary (p38α: −22.78 % and p38β: −27.41 %). Likewise, the MKK3 and MKK6 phosphorylation increased considerably (MKK3: 28.19 % and MKK6: 22.59 %) in SW-1353 cells, and decreased in Cal-78 cells (MKK3: −26.75 % and MKK6: −18.87 %). The same picture is given for members of the Akt family: a down-regulation for the Cal-78 cells (Akt1: −23.34 %, Akt2: −16.70 %, and Akt3: −20.13 %) and an augmentation when tested in SW1353 cells (Akt1: 12.85 %, Akt2: 19.29 %, and Akt3: 9.23 %) (Fig. 4a).

Western blot analysis confirmed the results from the proteome profiler phospho-MAPK array (Fig. 4b). Phosphorylation of Akt (pAkt) and p38 (pp38) were upregulated after the treatment with different concentrations of diacerein in SW-1353 cells, whereas, only minor changes can be observed in the Cal-78 cells. The used phospho-Akt antibody detects endogenous levels of Akt1, Akt2, and Akt3 only when phosphorylated at Ser473. The used p38 MAPK antibody detects endogenous levels of total p38α, -β and -γ MAPK protein. In addition, the expression of p53 was diminished in Cal-78 cells. The cross-linking of interesting genes of cell cycle regulation and MAPK pathways is given in Fig. 4c using IPA (Ingenuity Pathway Analysis) software.

### Diacerein did not induce apoptosis

**Fig. 5:**
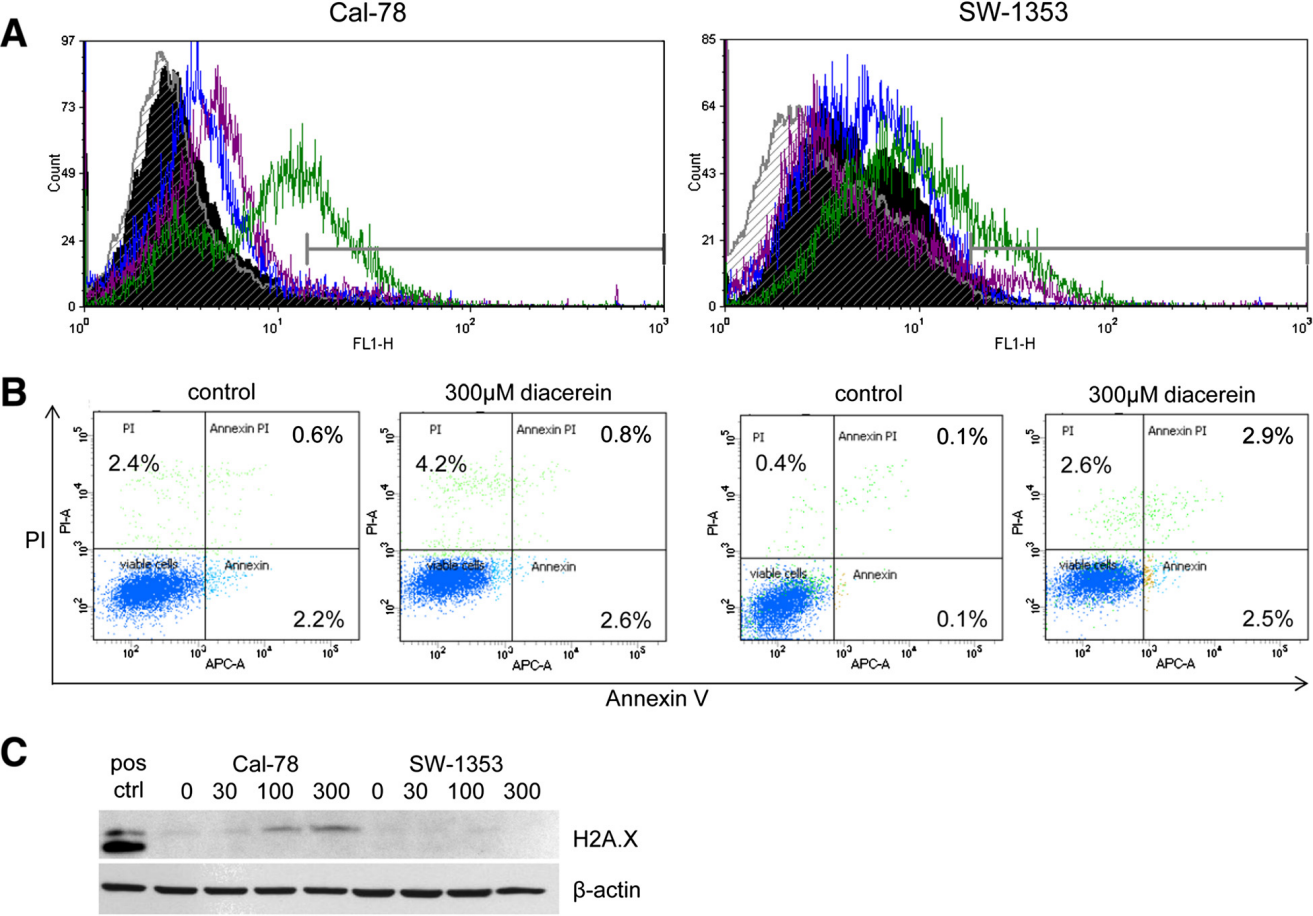
Cleaved caspase-3 and Annexin V/PI apoptosis assays. **a** Cal-78 and SW-1353 cells were treated with 300 μM diacerein. Cleavage of caspase-3 was detected after 48 h by flow cytometry. The y-axis denotes cell counts and the x-axis represents fluorescence intensity of APC antibody. Black filled histogram represents untreated control cells, striated histogram represents 30 μM diacerein, blue lanes showed 100 μM diacerein, and margenta lanes showed 300 μM diacerein treated cells. Green lanes represented the Staurosporin positive control. **b** Likewise, AnnexinV/PI staining under the influence of 300 μM diacerein for 48 h confirmed the lack of apoptosis induction. **c** In Cal-78 cells, the DNA damage marker H2AX was marginally increased. β-actin was used as loading control

Apoptosis induction was investigated by flow cytometric analysis of caspase-3 cleavage (Fig. 5a) and Annexin V/PI staining (Fig. 5b). Figure 4a shows the cleaved caspase-3 measurements after a 48 h exposure of the cells to 30, 100, and 300 μM diacerein. The flow cytometric analysis histograms represent untreated cells (black filled) versus 30 μM diacerein (striated lines), 100 μM diacerein (blue lanes), and 300 μM diacerein (margenta lanes) treated cells. Green lanes represent the Staurosporin positive control. In both cell lines only minimal caspase-3 cleavage could be detected, worth mentioning is the minor dose response detectable for the curve shift to the right within Cal-78 cells. Although, induction of apoptosis was further verified by Annexin V/PI staining, in accordance with the caspase-3 data, chondrosarcoma cells did not elicit an increase in Annexin positive cells. Studying the phospho-Histone H2A.X DNA damage marker, a slight concentration dependent increase in expression was detected for Cal-78 cells under the treatment with diacerein, while the SW-1353 cells did not show a response at the H2A.X expression level (Fig. 5c). These sets of data imply that diacerein do not significantly influence the induction of apoptosis.

## Discussion

Chondrosarcoma is characterized for its lack of response to conventional cytotoxic chemotherapy, propensity for developing lung metastases, and poor survival. Therefore research within the field of development and expansion of new treatment options is of particular priority.

The applied concentration of 100 μM diacerein reduced the cell viability of human chondrocytes by 20 % and accordingly induced a decrease in cell growth, while these two observations point to the fact that diacerein modulates cellular physiology [6]. Rhein, the metabolite of diacerein, has been demonstrated to induce anti-catabolic and anti-proliferative effects on chondrocytes stimulated by IL-1β at similar concentrations. Both effects were interpreted by alterations in cell cycle regulation and not by action of apoptosis [17]. However, the effect on chondrosarcoma has not been examined yet.

In initial experiments we showed, that in two different chondrosarcoma cell lines diacerein decreased the cell viability and the cell proliferation in a dose dependent manner. To identify the mechanism behind, within this study we reviewed the effect of diacerein on cell cycle distribution, the expression of cell cycle checkpoint proteins, the mitogen-activated protein kinases (MAPKs) pathways, and the induction of apoptosis in SW-1353 and Cal-78 cell lines.

In eukaryotes, the cell cycle is regulated by cyclins and cyclin-dependent kinases (CDKs). Cell cycle checkpoints enable cellular repair or may result in the activation of apoptosis signalling, if the cellular damages are significantly intense to be repaired properly [18, 19]. In particular, cyclin B and CDK1 proteins participate in the regulation of the progression of G2/M phase [20]. It is widely known that cells are blocked in the G2/M phase during DNA damage, and cells are more susceptible to the cytotoxic effects of radiotherapy in the G2/M phase [21]. Increasing induced G2/M phase arrest allows cell death which may be a useful strategy in cancer therapeutics [22].

The results of FACS analysis of SW-1353 cells treated with diverse concentrations of diacerein showed a decrease in the number of cells in G1 and S phase which was accompanied by a significant increase of the number of G2/M phase cells, strongly indicating a classical G2/M arrest. Interestingly, in the Cal-78 cell line only small changes in cell cycle distribution could be detected, a fact possibly ascribed to the differences in the origin of the two cell lines. SW-1353 cells were obtained from a primary grade II chondrosarcoma of the right humerus, whereas the Cal-78 cells were established from the recurrence of a dedifferentiated chondrosarcoma (grade III) of the muscle.

We further explored the effect of diacerein on the key regulators in the cell cycle checkpoints including CDK1, CDK2, and cyclin B1 in Cal-78 and SW-1353 cells. The CDK1-cyclin B1 complex is pivotal in regulating the G2/M phase transition and mitosis. We observed a significant decrease in the mRNA and protein expression levels of cyclin B1 and CDK1 in SW-1353, whereas in Cal-78 cells only the protein expression of cyclin B1 was significantly affected. The diacerein induced down-regulation of the cyclin B1-CDK1 complex might explain the observed reduction in cell growth in chondrosarcoma cells. Cyclin B1 is responsible for the transition of the cell from the G2 to the M phase but changes to a disruption in cancer cells where overexpression of cyclin B1 can lead to uncontrolled cell growth [19]. In addition both cell lines feature a significant reduction of CDK2 expression verified at the RNA and protein level, respectively. Altogether we are able to postulate, that diacerein influences cell cycle of chondrosarcoma cells, whereas the extent of the expression level of regulatory proteins involved appear to depend on the source of the cells. In our study Cal-78 cells are obvious more insensitive to diacerein regarding the cell cycle controls than the SW-1353 cells.

MAPKs are signaling components that are important in converting extracellular stimuli into a wide range of cellular responses. Signaling network is increasingly important for our comprehension of cell proliferation. The p38 MAPK as an important stress kinase is involved in the regulation of inflammation, cell growth and differentiation, cell cycle, and cell death [23]. Four isoforms of p38, known as p38α, p38β, p38γ, and p38δ have been identified, which can all be phosphorylated by the MAPK kinase MKK6 (SKK3) and MKK3, respectively [24]. p38 can negatively regulate cell cycle progression both at the G1/S and the G2/M transitions by several mechanisms, including the down-regulation of cyclins, up-regulation of CDK inhibitors and modulation of the tumor suppressor p53 [25]. Moreover, we also provided evidence about the existence of a crosstalk between the p38 MAPK and Akt/mTOR signaling pathways in SW-1353 cells exposed to diacerein. In the present study, we have observed that diacerein treatment significantly increases the phosphorylation of p38α and p38β MAPKs in SW-1353, whereas Cal-78 shows a decrease of these p38-MAPKs. These observations are in accordance with our cell cycle FACS and protein expression data. The serine-threonine Akt kinase family is well-known as crucial regulators of cell survival, proliferation, metabolism, and migration [26]. Deregulation of Akt kinases is frequently associated with human diseases such as cancer [27]. It has been reported previously that Akt activity is high in the G2/M phase of the cell cycle in epithelial cells [28]. Akt activity protects cells from apoptosis during the G2/M transition and is necessary for efficient changeover to mitosis during unperturbed cell cycles [29]. In addition to its role in cell cycle progression, Akt-mediated phosphorylation and cytoplasmic translocation of CDK2 is also important for apoptosis induced by stresses such as methotrexate and docetaxel [30]. Therefore, an increased phosphorylation of Akt1, Akt2, and Akt 3 in the SW-1353 cells after diacerein treatment explained the G2/M phase arrest.

These observed growth-inhibiting characteristics of higher concentrations of diacerein might implicate a therapeutic benefit for the treatment of chondrosarcoma. To elucidate the apoptotic potential of diacerein on chondrosarcoma cells, we have performed two different apoptosis assays. Diacerein treatment showed no apoptotic induction, neither in the Cal-78 nor in the SW-1353 cell line.

## Conclusion

Our results demonstrate for the first time that diacerein decreased the viability of human chondrosarcoma cells and induces G2/M cell cycle arrest by CDK1/cyclin B1 down-regulation. In summary, our findings strongly support diacerein as an interesting target for further investigation and development of novel therapeutics in sarcoma research.

## Abbreviations

ACTB: β-actin
CDK: Cyclin-dependent kinase
CI: Cell index
ERK1/2: Extracellular signal-regulated kinases 1/2
GAPDH: Glyceraldehyde 3-phosphate dehydrogenase
HPRT-1: Hypoxanthine phosphoribosyl-transferase
JNK1-3: c-Jun N-terminal kinases 1-3
MAPKs: Mitogen-activated protein kinases
OA: Osteoarthritis
RNA: Ribonucleic acid
RT-PCR: Reverse transcription polymerase chain reaction
SYSADOA: Symptomatic slow acting drug in osteoarthritis
